# A Novel Ensemble Method for Imbalanced Data Learning: Bagging of Extrapolation-SMOTE SVM

**DOI:** 10.1155/2017/1827016

**Published:** 2017-01-30

**Authors:** Qi Wang, ZhiHao Luo, JinCai Huang, YangHe Feng, Zhong Liu

**Affiliations:** Science and Technology on Information Systems Engineering Laboratory, College of Information System and Management, National University of Defense Technology, Changsha, Hunan, China

## Abstract

Class imbalance ubiquitously exists in real life, which has attracted much interest from various domains. Direct learning from imbalanced dataset may pose unsatisfying results overfocusing on the accuracy of identification and deriving a suboptimal model. Various methodologies have been developed in tackling this problem including sampling, cost-sensitive, and other hybrid ones. However, the samples near the decision boundary which contain more discriminative information should be valued and the skew of the boundary would be corrected by constructing synthetic samples. Inspired by the truth and sense of geometry, we designed a new synthetic minority oversampling technique to incorporate the borderline information. What is more, ensemble model always tends to capture more complicated and robust decision boundary in practice. Taking these factors into considerations, a novel ensemble method, called Bagging of Extrapolation Borderline-SMOTE SVM (BEBS), has been proposed in dealing with imbalanced data learning (IDL) problems. Experiments on open access datasets showed significant superior performance using our model and a persuasive and intuitive explanation behind the method was illustrated. As far as we know, this is the first model combining ensemble of SVMs with borderline information for solving such condition.

## 1. Introduction

In the domain of machine learning, data is essential for the model's training. However, the distribution of two classes behaves in extremely imbalanced way and the circumstance is quite ubiquitous in real life [1]. In this paper, we concentrate on the binary classification problems when there is extremely imbalanced distribution in two classes; that is, one class for training severely outnumbers the other.

With the neglect of the class imbalanced distribution, traditional algorithms for binary classification tend to perform badly on the dataset [2, 3], leading to the unsatisfied suboptimal result [4–7] that the majority class can be well identified while the minority is reverse. One of the reasons accounting for this is that the imbalanced distribution as prior information in many instances has a strong impact on final discrimination [8]. Now let us consider a special scenario in which the majority class amounts to the percentage of 99. In such case, an ordinary classifier that assigns any example to the majority class label would still achieve the accuracy of 99% [9]. However, due to the low recall ratio for the minority, such extreme result is not what we have desired. The phenomena are quite crucial and nontrivial in several circumstances, such as identification of network intrusion [10, 11], medical diagnosis of type 2 diabetes [12], oil spills detection from satellite radar images [13], finding of financial fraud transactions [14], and bioinformatics [15]. Another fact which cannot be neglected is that in most of the binary classification problems, the minority class is what we really care about rather than the majority [3], especially when the cost is expensive for the failure in recognizing the minority ones.

Numbers of algorithms have been designed for relieving the consequences of the imbalanced data. From the perspective of the strategy, these methods can be categorized as three mainstream types. In the algorithm level, the adjustment of the weights of errors in the loss function, also called cost-sensitive learning, is a direct way to reduce the impact of imbalance. Cost matrix which measures different penalties for misclassification is critical for the improvement on the performance. Another way to tune the penalties was rooted in adaptive boosting [16] and some algorithms, for example, Ada-cost [17] and cost-sensitive boosting [18], were implemented for the learning task. In the sampling level, the easiest way is to randomly sample the training data from the whole training dataset in the way that different classes of data are sampled in appropriate ratios to balance the proportions in classes. Random sampling tended to be overfitting if the sampling ratios were not properly modulated [3]. Repeated sampling was easier to implement but hard to adjust efficiently, so Provost [9] thought undersampling was proper for the larger training dataset while the synthetic samples were constructed for the less sample cases. Estabrooks et al. [2] combined different repeated sampling methods to offer a better scheme of modulation. Another popular method was the synthetic minority oversampling technique (SMOTE) proposed by Chawla et al. [19] and the core idea was to construct the synthetic minority samples through the interpolation between minority training data and its *k*-nearest neighborhoods. Han et al. [3] paid more attention to the samples near the decision borderline and combined the SMOTE to acquire the Borderline-SMOTE. Apart from the above sampling methods, clustering-based resampling algorithm [20] and SMOTE-Boost algorithm [21] were also designed for the imbalanced cases. Furthermore, fewer researchers focused on how the sampling methodologies impact on the learning performance but the reason why sampling methods boost the linear discriminative performance was given by Xue and Hall [6].

In addition to the algorithm and sampling based methods, other researchers proposed several practical and popular methods with excellent performance from the hybrid view. With the help of SMOTE on the boundary samples and the adjustment of kernel matrix, Wu and Chang [22] integrated the prior information of distribution of imbalance with SVM to obtain the kernel boundary alignment algorithm. Chawla et al. [4] noticed the great influence of feature selection and Maldonado et al. [23] did some research on various ways of feature selection and put forward backward elimination feature selection process for SVM's dealing with the IDL problem. Two-stage cost-sensitive learning was employed in the NASA imbalanced data and the researcher designed cost-sensitive rules for both feature selection and the classification stage [24]. Rather than former over- or undersampling methods, Sun et al. [25] utilized random partition and clustering tricks to obtain some balanced datasets for training various classifiers and combined them according to some rules. Bhowan et al. made use of genetic programming to construct types of fitness functions and exploit the multiobjective optimization for combining the classifiers [5]. Easy ensemble and balance cascade [26] were two superior algorithms, which made use of ensemble models learned in an undersampling way.

Quite different from the above-mentioned methods, we would propose a novel ensemble algorithm, in which an efficient sampling method was developed for IDL problems. Necessarily, similar works are summarized as follows. In the preprocessing process, Batuwita and Palade [27] screened some informative examples closer to the class boundary captured by SVM and downscaled the resampling of other samples to reduce the time complexity of training SVM with performance maintained. For the binary imbalanced data, Wu and Chang implemented boundary alignments using kernel tricks to relieve the offset of decision boundary [28]. From the perspective of ensemble, there exist several elaborate reviews about ensemble models for the IDL [8, 29]. Specifically, López et al. [8] studied how six significant problems relating to the data intrinsic characteristics affected the performance of ensemble models for the IDL. Several superior ensemble models are based on boosting, such as EUSBoost [30], evolutionary undersampling boosting model in dealing with breast cancer malignancy classification [31]. Shi et al. made use of bagging technique on SVM to cope with P300 detection problem [32]. Our proposed framework also made use of those samples near the decision boundary detected by the SVM but in a more flexible way of sampling. We applied bagging technique to meta-SVM trained with data obtained from the sampling process. The final results show our model's effectiveness in dealing with the IDL problem.

## 2. Materials and Methods

**Figure 1:**
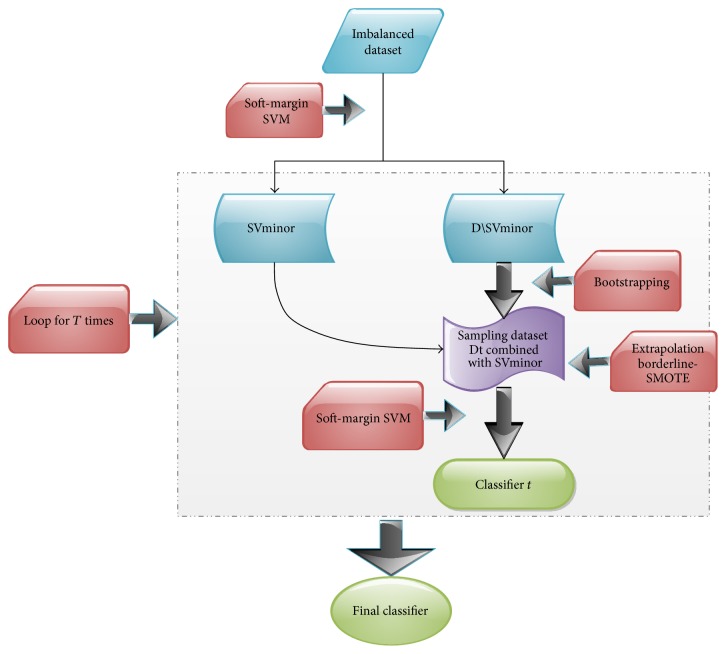
The flowchart of our proposed framework. *D* is the complete dataset and SVminor is the support vectors in the minority after the initialized SVM's detection.

Before our framework was introduced, some basic knowledge about models or techniques would be in a brief summary. In our framework, SVM works as metaclassifiers for ensemble and bootstrapping aggregation is a type of sampling technique to obtain various training dataset. Besides, we would illustrate SMOTE in a comprehensive way and induce our adaptive SMOTE technique. Flow chart about our framework is given in Figure 1.

### 2.1. Support Vector Machine: A Review

Support vector machine as one of the popular classifiers in binary classification has shown its state of art performance in engineering applications.

Given the labeled training set  *T* = {(**x**_**i**_, *y*_*i*_)∣*y*_*i*_ = 1  or  −1, *i* = 1 ⋯ *N*}, a naïve idea to learn a classifier is to characterize a hyperplane in the feature or transformed feature space of the input **x** that can separate two classes of training data as much as possible. Based on the statistical learning theory [33], SVM is recognized as the robust adaptation for the perceptron learning model [34].

Trick of feature transformation *ϕ* and soft-margin relaxation make SVM powerful for the detection of complex decision boundary and control the overfitting with the allowance for some samples' violating the support hyperplanes.

Here, we give an expression of SVM in the form of quadratic programming problem as follows:

(1) minW,b,ζ 12WT∗In∗W+C1NT∗ζs.t AWζb≥1N,

where *A* is the corresponding matrix

(2) $$\begin{array}{c} A=\left(\begin{array}{c} y_{1}\phi {\left(x_{1}\right)}^{T},\epsilon_{1},1\\ y_{2}\phi {\left(x_{2}\right)}^{T},\epsilon_{2},1\\ \vdots \\ y_{N}\phi {\left(x_{N}\right)}^{T},\epsilon_{N},1 \end{array}\right). \end{array}$$

*N*-dimension unit vector *ϵ*_*k*_ with *k*th coordinate equals 1.

The slacked vector of variables  *ζ* = (*ζ*_1_, *ζ*_2_,…,*ζ*_*N*_)^*T*^ measures the extent to samples' violating the support hyperplanes. The primal problem can be converted to the dual one by solving the Karush-Kuhn-Tucker optimal functions derived from the Lagrange equation [35].

Finally, the discriminative function is obtained in the form

(3) $$\begin{array}{c} f\left(\;x\;\right)=\mathrm{sign}\left(\;\underset{\alpha_{n}^{*}>0}{\sum }y_{n}\alpha_{n}^{*}\phi {\left(\;x_{n}\;\right)}^{T}*\phi \left(\;x\;\right)+b\;\right), \end{array}$$

where *α*_*n*_^*∗*^ is the Lagrange multiplier corresponding to the sample satisfying Karush-Kuhn-Tucker (KKT) optimal conditions.

Specifically, when the inner product of the transformed feature vectors can be applied with the kernel methods, the process of computation is efficient:

(4) $$\begin{array}{c} \phi {\left(\;x_{n}\;\right)}^{T}*\phi \left(\;x_{m}\;\right)=K\left(\;x_{n},x_{m}\;\right). \end{array}$$

Furthermore, only a small proportion of the training data corresponding to positive Lagrange multipliers called support vectors are useful for the final decision, so the representation of the classifier is rather sparse.

### 2.2. Ensemble Methods: A Review

Apparently one classifier may be severely affected when the training dataset cannot well characterize the actual underlying distribution or the presumed model is biased. The strategy of models' ensemble can avoid the one-sidedness originated from the training dataset and hypothesis, receiving a better capability of generalization. In another aspect, weaker classifiers are easier to obtain using simple criteria like stump and a strong classifier can be derived by combining multiple weaker classifiers [36]. In our proposed framework for the IDL, bagging technique was employed for developing various models.

Bootstrapping aggregation abbreviated as bagging constructs totally different classifiers based on the bootstrapping method. Bootstrapping technique samples each training example in the same probability with replacements.

The general bagging algorithm can be described as shown in Algorithm 1.

One of the most famous models motivated from bagging is random forest in which not only is the training data sampled in bootstrapping way, but the features for training are selected in random as well [37].  Table 1 provides a detailed process of sampling methods.

### 2.3. Bagging of Extrapolation Borderline-SMOTE SVM

#### 2.3.1. Extrapolation Borderline-SMOTE

For IDL, SMOTE [19] is a typical oversampling method with universal applications and the concrete process for generating the synthetic samples can be described as shown in Algorithm 2.

**Figure 2:**
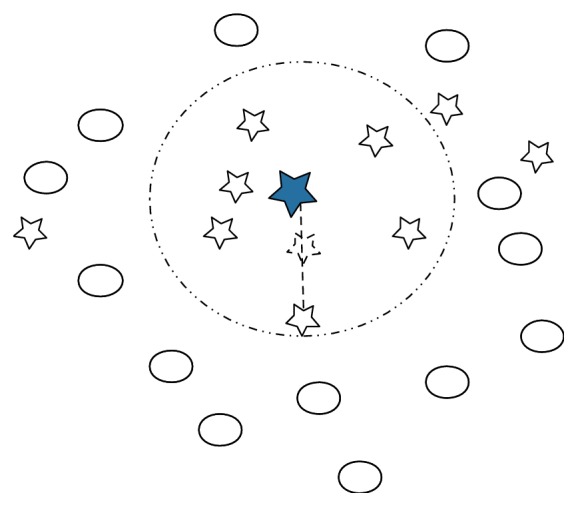
Manipulation of SMOTE. Stars stand for the minority and the solid star is selected for SMOTE. Circles are the majority. The selected sample randomly picks up one sample from it *k*-nearest neighbors to interpolate a synthetic sample labeled as the minority.

Generating some synthetic samples for the minority in an interpolation way is demonstrated to be effective for relieving the extent of imbalance and lifting performance. However, it seems that samples near the decision border overweigh the remaining ones in decision-making. Borderline-SMOTE [3] operates on samples near the decision border using SMOTE technique.  Figure 2 displays the interpolation method to generate synthetic samples.

**Figure 3:**
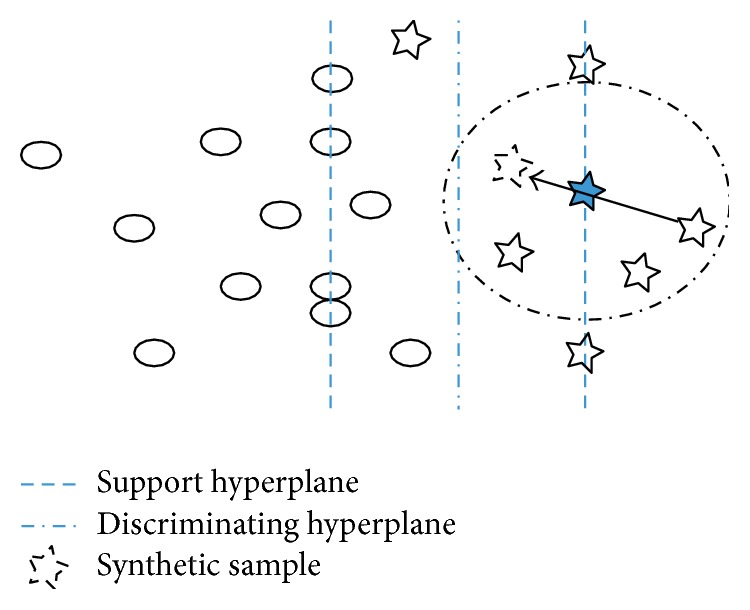
The effect of Extrapolation Borderline-SMOTE. X with no frame is the sample belonging to the minority after Extrapolation Borderline-SMOTE. A synthetic sample labeling to the minority explores towards the actual boundary and it seldom violates the original decision boundary with the help of *δ*.

However, the interpolation between samples used in SMOTE or Borderline-SMOTE restricts the ability of exploring towards the actual boundary. As we would make use of ensemble SVMs, samples near decision boundary can be roughly characterized from support hyperplane learned by the first SVM. Taking this into consideration, a novel synthetic minority oversampling method is proposed as shown in Algorithm 3 and Figure 3 describes our ideology.

Here 1/‖*w*‖_2_ is the distance from support hyperplane to decision hyperplane corresponding to the first SVM learned from the imbalance training dataset.

It is obvious that the synthetic minor sample tends to correct the skew finely and the extrapolation works to detect the decision boundary when *x*_*i*_^~^ belongs to the inner side of support hyperplane just as Figure 3 indicates.

#### 2.3.2. Bagging of Extrapolation Borderline-SMOTE SVM

Ensemble methods can effectively enhance model's capability of generalization. Here, a novel ensemble method for solving IDL problems is proposed called Bagging of Extrapolation Borderline-SMOTE SVMs (BEBS).

For SVM, it is noted that support vectors with positive Lagrange multipliers decide the final discriminative boundary. So we employ Extrapolation Borderline-SMOTE to the support vectors belonging to the minority for relieving the imbalance level.

The whole ideas about BEBS can be elucidated as follows. The original support vectors containing borderline information are roughly identified through the base SVM which is learned from the imbalance dataset  *D*. During the initialization process, a proper kernel and hyperparameter *C* are chosen through cross-validation in which G-means is chosen as the optimal metrics. Then the original support vectors belonging to the minority are marked as SV_0_ = {*x*_SV_*i*__^(0)^} and a novel dataset *D*^~^ = *D*∖SV_0_  for further bootstrapping is constructed by removing  SV_0_. Bootstrapping is performed on *D*^~^  in *K*-turns and each sampling result *D*_*t*_^~^ is in the scale of |*D*^~^|. Furthermore, aggregating datasets of *D*_*t*_^~^and SV_0_ are operated by Extrapolation Borderline-SMOTE. After that, the merged datasets with new synthetic samples are used for meta-SVM's training and the original data which are not sampled work as validation sets for tuning parameters. Finally, *K* SVMs are aggregated in the same weight to form the ensemble classifier (see Algorithm 4).

Specifically, default parameters in our model were initially set as *α* = 0.5, *K* = 100  and the following experiments shared the same parameters.

#### 2.3.3. The Intuition behind BEBS

The core idea of BEBS is to aggregate various SVMs which revise the initial decision boundary by constructing synthetic minority samples towards the correct direction. These synthetic samples are presumed to well characterize the actual decision boundary. The SVMs' variance originates from two sources. One is the random selection from SV_0_ with the sampling ratio  *α*% and the other originates from training sets' difference due to bootstrapping manipulation. Besides, training data not sampled in a trek of bootstrapping is exploited as the validation set for exploring a better hyperparameter; just see Table 1. All of these heuristic tactics were taken to enhance the model's generalization.

## 3. Results and Discussion

### 3.1. Experimental Settings and Metrics

Datasets for experiments are chosen from UCI machine learning repository [38] and most of them are quite imbalanced. Here we only cope with binary classification problems, so one class is labeled as the minority while the rest merge as the majority in multiclass cases which is similar to other researchers' preprocess [25, 39, 40].  Table 2 shows the detailed information about the dataset including sample capacity, the number of attributes, the numbers of the minority samples and majority samples, and the imbalance ratio. The imbalance ratio is defined as the result of the cardinality of the majority dividing the cardinality of the minority, which may severely influence the performance of classifiers.

Traditional ensemble methods like AdaBoostM1 and random forest are chosen for comparison as well. Further illustration that should be noticed is that both AdaBoostM1 and random forest can be considered as techniques relieving the imbalance due to the weight-adjustment mechanism by error in AdaBoostM1 and out-of-bag performance monitored in random forest. We also validated imbalance effect on original SVM. Some state-of-the-art and commonly used algorithms, including random undersampling, random oversampling, SMOTE, and SMOTE-ENN [41] were performed on the above-mentioned dataset as well, all of which would demonstrate the effectiveness of the novel proposed algorithm. Besides, random undersampling, random oversampling, SMOTE, and SMOTE-ENN were combined with SVM for further classification.

In problems of binary classification, confusion matrix offers an intuitive measure for evaluating classifier's performance. As illustrated in Table 3, FN is the number of samples identified as negative ones by mistake and the rest can be understood similarly.

The accuracy of the classifier is defined as

(5) $$\begin{array}{c} Acc=\frac{TP+TN}{TP+FN+FP+TN}. \end{array}$$

For the problem of IDL, the accuracy is not persuasive for evaluation as depicted before. One of the most frequently used evaluation criteria for IDL is G-means which penalizes the biased model strictly. G-means is an index averaging geometrically the recall ratios of two classes.

(6) $$\begin{array}{c} GM={\left(\;\frac{TP}{TP+FN}*\frac{TN}{FP+TN}\;\right)}^{1/2}. \end{array}$$

It is obvious that only when both of the recall ratios stay at higher level can the G-means receives better value. So the G-means can be considered to be the trade-off between the accuracy and the recall ratio.

Another evaluation index penalizing the imbalanced effect is *F*-score defined as

(7) $$\begin{array}{c} F_{\beta }=\frac{\left(1+\beta^{2}\right)\left(\left(TP/\left(TP+FP\right)\right)*\left(TP/\left(TP+FN\right)\right)\right)}{\left(\beta^{2}TP/\left(TP+FP\right)\right)+\left(TP/\left(TP+FN\right)\right)}. \end{array}$$

Harmonic average is applied in the index and the parameter *β* controls the extent for penalization. Here *β* is selected as 1.

*F*-score shows similar performance and shares consistency with G-means in our experimental findings, but it averages the precision and recall ratio of one class in essence.

Besides, the precision of the minority in one classifier also plays a crucial role in IDL and most of cases show its significance just as the introduction has described. So the precision was taken into consideration during evaluating process. The precision for the positive is denoted as

(8) $$\begin{array}{c} Precision=\frac{TP}{TP+FP}. \end{array}$$

To obtain a robust result for evaluation, we picked up risk minimization as the criteria in which the minimum metrics of binary class were defined as the corresponding result. Taking precision for an instance, though precisions of both classes can be computed during testing process, the smaller one was selected, just as follows:

(9) $$\begin{array}{c} Precision≔\underset{i}{\min}\;Precision\left(\;class\;\;i\;\right). \end{array}$$

### 3.2. Results Analysis

#### 3.2.1. Performance Analysis

**Figure 4:**
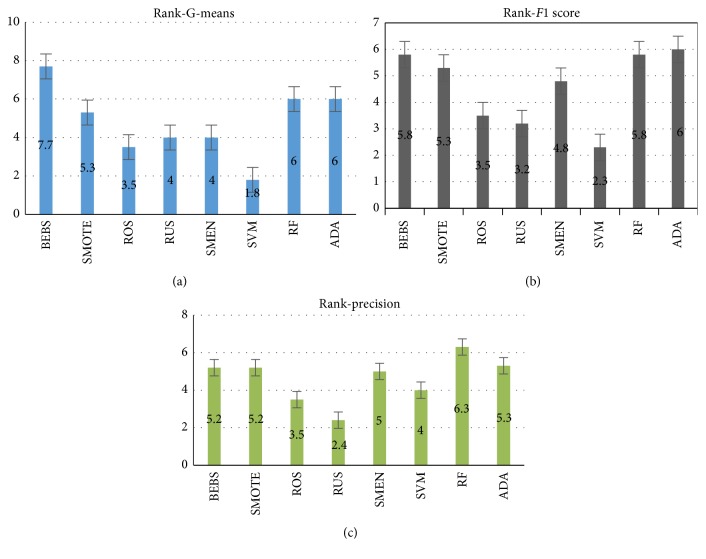
The average rankings of different algorithms on six datasets from three aspects. Rankings are integer scores as 8, 7, 6, 5, 4, 3, 2, and 1 assigned to each algorithm according to the performance.

We, respectively, averaged the results of G-means, *F*1-score, and precision in 10 independent turns.  Table 4 was the final results on various dataset and the top 3 ones in each line were labeled with bold. A direct conclusion drawn from the table was BEBS, random forest, and AdaBoostM1 located in dominating board most of time and behaving stably in three metrics. Some reason accounting for this was that the requirements of careful adaptation about parameters for all the other sampling algorithms seemed crucial. However, the original SVM received worse results on dataset of Fertility, Pima, Segmentation 1, Segmentation 3, and *F*1-score in Parkinson was rather low. Such phenomenon verified the explanation about skew of SVM in imbalanced case. It was evident that random oversampling, random undersampling, and SMOTE-ENN were sensitive to the datasets because all of them needed parameters of manual setting according to specific case rather than adapting automatically. SMOTE outperformed these three methods but was less efficient than our proposed BEBS. Obviously, BEBS which performed well in three metrics stably benefited from both the intuitive extrapolation-SMOTE method involving boundary information and randomness from bootstrapping technique. To offer a more direct cognition, we ranked the performance of methods on testing sets in decreasing order from the perspective of G-means, *F*1-score, and precision. The average ranks of all algorithms on 6 datasets were shown in Figure 4. Also, taking generalization and performance into consideration, random forest and AdaBoostM1 were still worthwhile to make a trial with no additional information.

Specifically, with the help of SPSS [42], we carried out Student's paired *t*-test, in which confidence interval of difference was set as 95%, to check the significance of the 10 independent results in comparison. 10 independent results were compared in the form of BEBS versus some other algorithm. Since seven models were chosen for comparison, seven statistical testing results were obtained on each dataset. We looped such process in metrics G-means, *F*-scores, and precision on each dataset. Furthermore, the seven pairs of the testing have three possible results, respectively, significantly weaker than BEBS and tie, significantly stronger than BEBS. Precise explanation about the result of tie is when the average of 10 independent results in some metrics on the dataset using model *A* is higher or lower than the BEBS but is not significant from the analysis of pair *t*-test, we directly attribute the reason behind difference to the randomness rather than the mechanism of models and label the paired comparison as tie. The label win means our BEBS's average of results not only outperforms the comparison model but also passes the hypothesis test. The same is the loss. Combined with Table 4, the results of significance testing were finally mapped into the 3-element tuple in the form of win∖tie∖loss. Then we counted the frequencies for win, tie, and loss in 7-paired comparison. So the computational results were arranged in the Table 5.

From Table 5, some obvious conclusions were drawn as follows. From the perspective of G-means, about 76.2% comparison results shown BEBS significantly outperformed other models which was computed as the total number of paired comparisons 42 divided by the number of win counts on the whole dataset 32. The ratio of no loss to others in *F*-scores occupied approximately 83.3% and at the same time 64.3% proportion of the total number of paired comparisons indicated superior results using BEBS compared with others significantly. For the precision, only 4.8% of the total counts were significantly poorer than some other models though the proportion of the tie counts maintained about 38.1%. In all, BEBS produced better results after a series of experiments and statistical testing process. The next part would do some research on the stability of the BEBS and some sensitivity analysis experiments were carried out.

#### 3.2.2. Sensitivity Analysis

It is noticed that our proposed algorithm BEBS contains two crucial hyperparameters to tune, that is, the number of metaclassifiers *K* and the oversampling ratio for Extrapolation Borderline-SMOTE  *α*%. Regardless of variations on dataset in the former experiments, the hyperparameters were consistently set as the fixed values *K* = 100 and sampling ratio = 0.5. The performance ought to be influenced when such parameters are violated. To investigate the robustness of the BEBS, we performed BEBS on the prepared dataset given a tunable range of hyperparameters. As suggested before, G-means is capable of well characterizing the fair results by imposing the penalization on the imbalance consequence. Here sensitivity analysis towards two hyperparameters was carried out and G-means was the objective we concentrated on.

**Figure 5:**
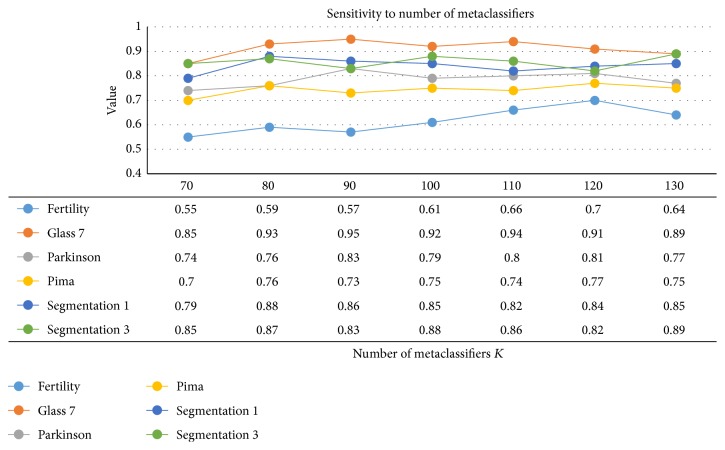
Sensitivity analysis on the number of metaclassifiers. The value in the table (except the first row) is the averaged results.

With the sampling ratio fixed as 0.5, we ranged the number of metaclassifiers *K* in the interval [70,130] at the step length 10 and averaged the 10 independent results corresponding to the fixed parameters. As Figure 5 illustrated, the six polylines run steadily as *K* increased and the maximum of ranges of G-means values on six polylines was not larger than 0.15. The results suggested BEBS was not sensitive to the number of metaclassifiers at the range [70,130].

**Figure 6:**
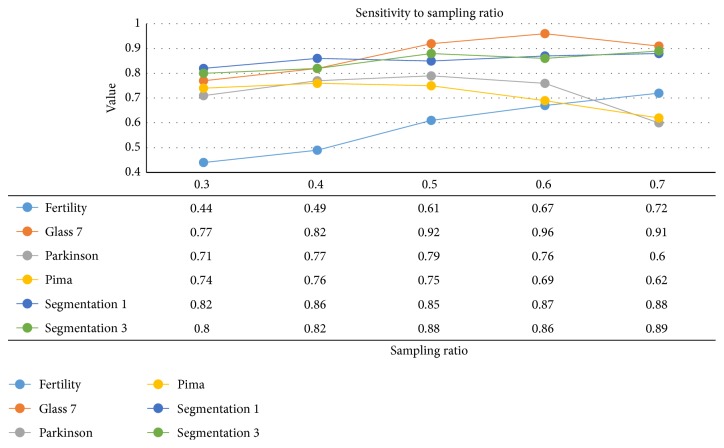
Sensitivity analysis on the sampling ratio. The value in the table (except the first row) is the averaged results.

Furthermore, we adapted the sampling ratio in a range of [0.3,0.7] at the step length 0.1 on the dataset while the number of metaclassifiers *K* was maintained as 100. The experimental results were shown in Figure 6. The points on polylines were acquired by averaging G-means values from 10 independent results given a set of parameters as well. An interesting fact lied in the fact that tendencies on Fertility, Glass 7, and Segmentation 3 were significant and performance was steadily enhanced when increasing the sampling ratio. The phenomenon may be attributed to the imbalance ratio of dataset. The imbalance ratios on these were not less than 6 from statistical information in Table 2. More synthetic minority samples tended to make contributions towards detecting the actual boundary. So a conclusion can be drawn that when the imbalance ratio retains a rather higher level, the sampling ratio should also adapt to relieve the overfitting circumstance. Results on Parkinson and Pima indicated declines when sampling ratio is higher than some thresholds, so higher sampling ratio on not extremely imbalanced dataset may do damage to the final performance. In total, BEBS seems sensitive to the Resampling Ratio and the imbalance ratio should be involved in a fine choice for the parameter.

## 4. Conclusions

In this paper, a novel ensemble method called BEBS was proposed for dealing with the IDL in binary case. The BEBS was framed by employing an adaptive sampling method Extrapolation Borderline-SMOTE and bootstrapping aggregation to the former imbalanced dataset. Such variant of SMOTE takes advantage of boundary information derived from the initial SVM and bagging's mechanism contributes to the relief of overfitting and promotes the capability of model's generalization. The decision boundary's skew towards the minority when using SVM can be revised with the help of synthetic samples. In our experiments, the results on each dataset run for ten times independently to ensure the effectiveness of hypothesis test and further statistical records show BEBS can significantly outperform some representative IDL algorithms in most of time. The sensitivity analysis illustrates the relation between scale of ensemble, sampling ratio, and performance, suggesting BEBS would be extensively enhanced after a proper adaptation according to imbalance ratio of dataset. Future research will summarize general relations between algorithms performance and other attributes like attributes' number and samples' cardinality. Multiclass imbalance cases [43] are also considered in the later mining tasks.

## Figures and Tables

**Algorithm 1:**
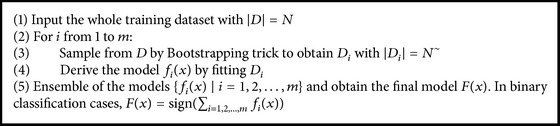
Bagging algorithm.

**Algorithm 2:**
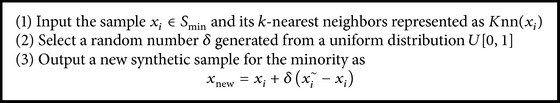
SMOTE algorithm.

**Algorithm 3:**
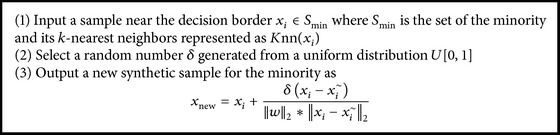
Extrapolation Borderline-SMOTE algorithm.

**Algorithm 4:**
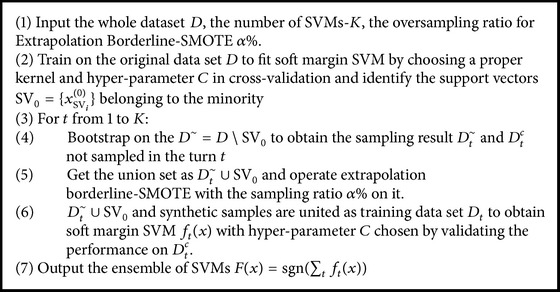
BEBS algorithm.

**Table 1:** The process of bootstrapping. The symbol √ marks the data which is sampled in the turn while ○ marks the data which is not sampled. Specifically, sets that are not sampled in each turn can be used as validation set for parameters' tuning.

Training dataset					
	*x*_1_	*x*_2_	*x*_3_	…	*x*_*N*_
1	√	○	√	…	√
2	○	√	√	…	
3	○	√	○	…	√
…	○	○	○	…	
*T* − 1	√	○	○	…	√
*T*	√	√	○	…	○

**Table 2:** The basic information about six datasets.

ID	Dataset	# attributes	# majority samples	# minority samples	IR
1	Fertility	10	88	12	7.3
2	Glass 7	10	185	29	6.3
3	Parkinson	23	147	48	3
4	Pima	8	500	268	1.8
5	Segmentation 1	19	180	30	6
6	Segmentation 3	19	180	30	6

**Table 3:** Confusion matrix.

	Predicted positive	Predicted negative
Actual positive	TP	FN
Actual negative	FP	TN

**Table 4:** The average results on various dataset using algorithms. ROS means random oversampling while RUS is random undersampling. SMEN is SMOTE-ENN. RF is random forest and ADA is AdaBoostM1.

		BEBS	SMOTE	ROS	RUS	SMEN	SVM	RF	ADA
Fertility	G-means	**0.613**	**0.521**	0.446	**0.532**	**0.532**	0	0.063	0.424
	*F*-scores	**0.285**	**0.233**	0.194	0.23	**0.242**	0	0.029	0.202
	Precision	**0.624**	0.159	0.128	0.145	**0.171**	0	0.05	**0.194**
Glass 7	G-means	**0.923**	**0.912**	0.902	0.906	0.891	0.873	**0.914**	0.906
	*F*-scores	0.736	**0.882**	**0.878**	0.845	**0.877**	0.857	0.876	0.835
	Precision	0.902	0.906	**0.932**	0.842	**0.914**	**0.96**	**0.914**	0.831
Parkinson	G-means	**0.792**	0.523	0.464	0.392	0.436	0.278	**0.791**	**0.776**
	*F*-scores	**0.63**	0.442	0.324	0.284	0.336	0.15	**0.729**	**0.681**
	Precision	0.671	0.727	0.692	0.278	0.735	**0.769**	**0.809**	**0.736**
Pima	G-means	**0.753**	0.071	0	0	0	0	**0.642**	**0.685**
	*F*-scores	**0.681**	0.011	0	0.02	0	0	**0.55**	**0.613**
	Precision	**0.72**	**0.654**	0	0.07	0	0	**0.666**	0.632
Segmentation 1	G-means	**0.856**	0.297	0	0.103	0.262	0	**0.873**	**0.921**
	*F*-scores	**0.552**	0.143	0	0.024	0.144	0	**0.85**	**0.87**
	Precision	0.731	**0.871**	0	0.143	**0.869**	0	**0.95**	0.85
Segmentation 3	G-means	**0.884**	0.261	0	0.072	0.193	0	**0.746**	**0.833**
	*F*-scores	**0.611**	0.16	0	0.021	0.094	0	**0.665**	**0.762**
	Precision	**0.792**	0.767	0	0.143	0.667	0	**0.825**	**0.841**

**Table 5:** Win∖tie∖loss counts on results of paired *t*-test.

	Fertility	Glass 7	Parkinson	Pima	Segmentation 1	Segmentation 3
G-means	7∖0∖0	1∖6∖0	5∖2∖0	7∖0∖0	6∖1∖0	6∖1∖0
*F*-score	5∖2∖0	0∖2∖5	5∖1∖1	7∖0∖0	5∖2∖0	5∖1∖1
Precision	7∖0∖0	2∖5∖0	1∖4∖2	7∖0∖0	3∖4∖0	4∖3∖0

## References

[1] He H., Garcia E. A. (2009). Learning from imbalanced data. *IEEE Transactions on Knowledge and Data Engineering*.

[2] Estabrooks A., Jo T., Japkowicz N. (2004). A multiple resampling method for learning from imbalanced data sets. *Computational Intelligence*.

[3] Han H., Wang W.-Y., Mao B.-H. (2005). Borderline-SMOTE: a new over-sampling method in imbalanced data sets learning. *Advances in Intelligent Computing*.

[4] Chawla N. V., Japkowicz N., Kotcz A. (2004). Editorial: special issue on learning from imbalanced data sets. *ACM SIGKDD Explorations Newsletter*.

[5] Bhowan U., Johnston M., Zhang M. (2012). Developing new fitness functions in genetic programming for classification with unbalanced data. *IEEE Transactions on Systems, Man, and Cybernetics, Part B: Cybernetics*.

[6] Xue J.-H., Hall P. (2015). Why does rebalancing class-unbalanced data improve AUC for linear discriminant analysis?. *IEEE Transactions on Pattern Analysis and Machine Intelligence*.

[7] Batuwita R., Palade V. (2013). Class imbalance learning methods for support vector machines. *Imbalanced Learning: Foundations, Algorithms, and Applications*.

[8] López V., Fernández A., García S., Palade V., Herrera F. (2013). An insight into classification with imbalanced data: empirical results and current trends on using data intrinsic characteristics. *Information Sciences*.

[9] Provost F. Machine learning from imbalanced data sets 101.

[10] Pelayo L., Dick S. Applying novel resampling strategies to software defect prediction.

[11] Long J., Yin J.-P., Zhu E., Zhao W.-T. A novel active cost-sensitive learning method for intrusion detection.

[12] Zahirnia K., Teimouri M., Rahmani R., Salaq A. Diagnosis of type 2 diabetes using cost-sensitive learning.

[13] Kubat M., Holte R. C., Matwin S. (1998). Machine learning for the detection of oil spills in satellite radar images. *Machine Learning*.

[14] Fawcett T., Provost F. (1997). Adaptive fraud detection. *Data Mining and Knowledge Discovery*.

[15] Triguero I., del Río S., López V., Bacardit J., Benítez J. M., Herrera F. (2015). ROSEFW-RF: the winner algorithm for the ECBDL'14 big data competition: an extremely imbalanced big data bioinformatics problem. *Knowledge-Based Systems*.

[16] Freund Y., Schapire R. E. (1995). A decision-theoretic generalization of on-line learning and an application to boosting. *Lecture Notes in Computer Science (including subseries Lecture Notes in Artificial Intelligence and Lecture Notes in Bioinformatics)*.

[17] Fan W., Stolfo S. J., Zhang J., Chan P. K. AdaCost: misclassification cost-sensitive boosting.

[18] Masnadi-Shirazi H., Vasconcelos N. (2011). Cost-sensitive boosting. *IEEE Transactions on Pattern Analysis and Machine Intelligence*.

[19] Chawla N. V., Bowyer K. W., Hall L. O., Kegelmeyer W. P. (2002). SMOTE: synthetic minority over-sampling technique. *Journal of Artificial Intelligence Research*.

[20] Jo T., Japkowicz N. (2004). Class imbalances versus small disjuncts. *ACM SIGKDD Explorations Newsletter*.

[21] Chawla N. V., Lazarevic A., Hall L. O., Bowyer K. W. (2003). SMOTEBoost: improving prediction of the minority class in boosting. *Knowledge Discovery in Databases: PKDD 2003: 7th European Conference on Principles and Practice of Knowledge Discovery in Databases, Cavtat-Dubrovnik, Croatia, September 22–26, 2003. Proceedings*.

[22] Wu G., Chang E. Y. (2005). KBA: kernel boundary alignment considering imbalanced data distribution. *IEEE Transactions on Knowledge & Data Engineering*.

[23] Maldonado S., Weber R., Famili F. (2014). Feature selection for high-dimensional class-imbalanced data sets using Support Vector Machines. *Information Sciences*.

[24] Liu M., Miao L., Zhang D. (2014). Two-stage cost-sensitive learning for software defect prediction. *IEEE Transactions on Reliability*.

[25] Sun Z., Song Q., Zhu X., Sun H., Xu B., Zhou Y. (2015). A novel ensemble method for classifying imbalanced data. *Pattern Recognition*.

[26] Liu X.-Y., Wu J., Zhou Z.-H. (2009). Exploratory undersampling for class-imbalance learning. *IEEE Transactions on Systems, Man, and Cybernetics, Part B: Cybernetics*.

[27] Batuwita R., Palade V. Efficient resampling methods for training support vector machines with imbalanced datasets.

[28] Wu G., Chang E. Y. Class-boundary alignment for imbalanced dataset learning.

[29] Galar M., Fernandez A., Barrenechea E., Bustince H., Herrera F. (2012). A review on ensembles for the class imbalance problem: bagging-, boosting-, and hybrid-based approaches. *IEEE Transactions on Systems, Man, and Cybernetics, Part C: Applications and Reviews*.

[30] Galar M., Fernández A., Barrenechea E., Herrera F. (2013). EUSBoost: enhancing ensembles for highly imbalanced data-sets by evolutionary undersampling. *Pattern Recognition*.

[31] Krawczyk B., Galar M., Jeleń Ł., Herrera F. (2016). Evolutionary undersampling boosting for imbalanced classification of breast cancer malignancy. *Applied Soft Computing Journal*.

[32] Shi X., Xu G., Shen F., Zhao J. Solving the data imbalance problem of P300 detection via random under-sampling bagging SVMs.

[33] Vapnik V. N. (1998). *Statistical Learning Theory*.

[34] Rosenblatt F. (1958). The perceptron: a probabilistic model for information storage and organization in the brain. *Psychological Review*.

[35] Boyd S., Vandenberghe L. (2004). *Convex Optimization*.

[36] Zhou Z.-H. (2012). *Ensemble Methods: Foundations and Algorithms*.

[37] Liaw A., Wiener M. (2002). Classification and regression by randomForest. *R News*.

[38] Blake C., Merz C. J. (1998). *{UCI} Repository of Machine Learning Databases*.

[39] Zięba M., Tomczak J. M. (2015). Boosted SVM with active learning strategy for imbalanced data. *Soft Computing*.

[40] Xu Y., Yang Z., Zhang Y., Pan X., Wang L. (2016). A maximum margin and minimum volume hyper-spheres machine with pinball loss for imbalanced data classification. *Knowledge-Based Systems*.

[41] Batista G. E., Prati R. C., Monard M. C. (2004). A study of the behavior of several methods for balancing machine learning training data. *ACM SIGKDD Explorations Newsletter*.

[42] Norušis M. J. (1986). *SPSS/PC+ for the IBM PC/XT/AT*.

[43] Zhang Z., Krawczyk B., Garcìa S., Rosales-Pérez A., Herrera F. (2016). Empowering one-vs-one decomposition with ensemble learning for multi-class imbalanced data. *Knowledge-Based Systems*.

